# In silico high-throughput screening system for INMT activators in prostate cancer therapy

**DOI:** 10.3389/fphar.2026.1852736

**Published:** 2026-07-23

**Authors:** Haoyuan Zheng, Shilong Cao, Zhuoling Kong, Peng Xin, Jianbin Bi, Jianfeng Wang

**Affiliations:** 1 Department of Urology, First Hospital of China Medical University, Shenyang, China; 2 Institute of Urology, China Medical University, Shenyang, China

**Keywords:** apoptosis, castration-resistant prostate cancer, HTS, high throughput screening, INMT, prostate cancer

## Abstract

Prostate cancer (PCa) is one of the leading causes of cancer-related mortality in men, with castration-resistant prostate cancer (CRPC) posing significant therapeutic challenges due to drug resistance mediated by the androgen receptor (AR) signaling pathway. Indolethylamine-N-methyltransferase (INMT), a tumor-suppressive enzyme downregulated in CRPC, regulates pathways associated with apoptosis and proliferation. Leveraging a structure-based drug design strategy, this study aimed to identify novel INMT agonists for CRPC therapy. Virtual screening of the ChemDiv compound library (guided by the INMT crystal structure, PDB ID: 2A14), combined with molecular docking, MM/GBSA binding energy calculations, and molecular dynamics simulations, identified five candidate compounds. Among these, DMPP-4M stably bound to an allosteric site adjacent to the catalytic domain of INMT via hydrogen bonds, π-cation interactions, and hydrophobic forces. *In vitro* experiments demonstrated that DMPP-4M dose-dependently upregulated INMT expression, inhibited proliferation, and induced apoptosis in CRPC cell models (PC-3, 22RV1). The mechanism involved activation of the pro-apoptotic protein BAX, suppression of the anti-apoptotic protein Bcl-2, and upregulation of cleaved caspase-3 and PARP. Further mechanistic studies revealed that DMPP-4M-mediated INMT activation suppressed the activity of the TGF-β/Smad and Wnt/β-catenin signaling pathways. These findings suggest that DMPP-4M represents a promising INMT-targeted therapeutic agent, offering an AR-independent strategy for CRPC treatment. Subsequent structural optimization and *in vivo* experimental validation are required to advance its clinical translation potential.

## Introduction

Prostate cancer ranks as the second most prevalent malignancy and the second leading cause of cancer-related mortality in males worldwide, following lung cancer (Rebello et al., 2021). Androgen deprivation therapy (ADT) demonstrates initial efficacy in treating advanced and metastatic prostate cancer due to the critical role of androgen receptor (AR) signaling in mediating tumor survival and progression (Scott, 2018; Teo et al., 2019; Yamada and Beltran, 2021). However, a significant proportion of patients typically progress to castration-resistant prostate cancer (CRPC) within 2–3 years of treatment initiation (Desai et al., 2021; Wang et al., 2018). Despite the development of multiple therapeutic agents targeting CRPC, drug resistance inevitably emerges through various AR-dependent mechanisms (Matsumoto et al., 2013; Massie et al., 2011). These resistance pathways encompass AR gene amplification and/or mutational alterations, expression of AR splice variants, enhanced intratumoral androgen biosynthesis, and dysregulation of AR coactivators or corepressors (Li et al., 2013; Konda and Viswanathan, 2022; Hendriksen et al., 2006; Heinlein and Chang, 2004). Consequently, investigation of novel therapeutic targets and development of innovative agents against CRPC may offer valuable insights for developing therapeutic strategies against this treatment-resistant disease stage (Qi et al., 2021; Hsu et al., 2011).

Indolethylamine-N-methyltransferase (INMT), alternatively designated as aromatic alkylamine N-methyltransferase, indolamine N-methyltransferase, arylamine N-methyltransferase, thioether S-methyltransferase, amine N-methyltransferase (Dean, 2018), or nicotine N-methyltransferase, functions as a methyltransferase enzyme mediating the methylation process (Aghajanian and Marek, 1999). This catalytic activity involves transferring methyl groups from the universal methyl donor S-adenosyl-L-methionine (SAM) to specific substrate molecules (Grammenos and Barker, 2015; Axelrod, 1962). Indolethylamine-N-methyltransferase (INMT) facilitates detoxification of selenium compounds and modulates tryptophan metabolism through catalyzing N-methylation of tryptamine and structurally related derivatives (Herman et al., 1985; Barker et al., 2012). Our previous study has demonstrated that INMT serves as a promising prognostic biomarker for prostate cancer (Jianfeng et al., 2022). TCGA-PRAD analysis and validation in 30 pairs of clinical specimens showed that INMT is significantly downregulated in prostate cancer at both mRNA and protein levels, and its low expression correlates with lymph node metastasis (p < 0.001), higher Gleason scores (p = 0.024), elevated PSA (p = 0.012), and poorer disease-free survival (p = 0.005, HR = 0.58), suggesting a tumor-suppressive role. Mechanistically, INMT exerts its effects through regulation of MAPK, TGF-β, and Wnt/β-catenin signaling pathways—all of which can drive tumor progression independently of AR signaling, making INMT a potential AR-independent therapeutic target for CRPC. However, no pharmacological strategies are currently available to activate INMT for cancer therapy. In this study, we performed structure-based virtual screening to identify small-molecule INMT agonists and evaluated their anti-tumor efficacy in preclinical CRPC models.

## Materials and methods

### Molecular dynamics simulations

Molecular dynamics simulations were performed using GROMACS with docking-optimized complexes as initial configurations. The AMBER14SB force field and GAFF2 parameters were assigned to protein and ligand systems, respectively. Solvation was achieved by embedding the protein-ligand complex in a TIP3P water box with sodium counterions for charge neutralization. Electrostatic interactions were calculated using the Particle Mesh Ewald (PME) method with Verlet cutoff scheme and conjugate gradient optimization. Energy minimization (50,000 steps maximum) was conducted via steepest descent algorithm. Non-bonded interactions were truncated at 1.4 nm for both Coulombic and van der Waals potentials. Sequential equilibration included NVT (constant volume) and NPT (constant pressure) ensembles prior to 100-ns production simulations under standard conditions (300 K, 1 atm).

### Schrödinger

Computational workflows were conducted using Schrödinger software for protein preprocessing and docking grid generation. Pharmacophore models were developed for both a small molecule compound library and an INMT agonist. Virtual screening aligned the INMT agonist pharmacophore model with the ChemDiv database. A tiered protocol incorporating ADMET prediction and sequential high-throughput virtual screening (HTVS), standard-precision (SP), and extra-precision (XP) docking was executed. MMGBSA binding energy calculations prioritized the top five compounds for binding mode analysis. Detailed methodological procedures and corresponding outcomes are documented in the Results section.

### Virtual screening workflow and selection criteria

The ChemDiv compound library containing approximately 2,345,497 compounds was used for virtual screening. The tiered screening workflow employed specific selection criteria at each stage:

ADMET filtering: An initial ADMET filter was applied using the QikProp module to remove compounds with unfavorable drug-like properties. Only compounds satisfying Lipinski’s Rule of Five (molecular weight < 500 Da, logP < 5, hydrogen bond donors < 5, hydrogen bond acceptors < 10) and lacking reactive functional groups (e.g., aldehydes, Michael acceptors, epoxides) were retained for subsequent docking studies.

HTVS docking: High-throughput virtual screening (HTVS) was performed as the first docking stage. The top 10% of compounds by Glide HTVS score (approximately 233,585 compounds) were advanced to the next stage.

SP docking: Standard precision (SP) docking was then performed to further refine the hits. The top 10% of compounds from SP docking (approximately 23,358 compounds) were selected for extra-precision docking.

XP docking: Extra precision (XP) docking was carried out for the most accurate binding mode prediction. The top 10% of compounds by Glide XP score (approximately 2,338 compounds) were subjected to MM/GBSA binding free energy calculations.

MM/GBSA selection: Molecular Mechanics/Generalized Born Surface Area (MM/GBSA) calculations were performed to estimate binding free energies. Compounds with MM/GBSA ΔG_bind ≤ −30 kcal/mol were considered promising candidates. The final five compounds were selected based on a combination of favorable MM/GBSA scores (<−40 kcal/mol), optimal binding modes (including key hydrogen bond interactions and hydrophobic contacts with the allosteric site), and structural diversity among the top hits.

MMGBSA binding energy calculations prioritized the top five compounds for binding mode analysis.

### Reagents and cell lines

Human prostate cancer cell lines PC-3 (RRID:CVCL_0035) and 22RV1 (RRID:CVCL_1789) were obtained from the National Certified Cell Bank (Shanghai, China) in 2023. Cell line authentication was performed using short tandem repeat (STR) profiling, and the results showed ≥98% identity with the reference profiles, confirming no misidentification. All cells were confirmed to be mycoplasma-free by PCR assay before use in experiments. PC-3 cells were cultured in F12K medium (Gibco), while 22RV1 cells were maintained in RPMI 1640 (Gibco), both supplemented with 10% fetal bovine serum (FBS, Gibco) and 1% penicillin-streptomycin (Gibco), under standard culture conditions (37 °C, 5% CO_2_).

### CCK-8

PC-3 and 22RV1 cells were seeded in 96-well plates until reaching 80% confluency, followed by incubation with novel compounds for specified durations. CCK-8 reagent (Bimake, United States) was administered at 0.5 mg/mL to each well according to the manufacturer’s protocol. After 2-h incubation at 37 °C, cellular absorbance was quantified at 450 nm using a microplate reader (Model 680, Bio-Rad Laboratories).

### Western blot analysis

Protein extraction was performed using RIPA lysis buffer supplemented with protease and phosphatase inhibitors. Protein concentrations were quantified via bicinchoninic acid (BCA) assay (Beyotime Institute of Biotechnology). Samples (40 µg/lane) underwent electrophoretic separation on 10% SDS-PAGE gels (140 V, 60 min) followed by electrotransfer to PVDF membranes (350 mA, 90 min). Membranes were blocked with 5% non-fat milk in Tris-buffered saline containing 0.1% Tween-20 (TBST) for 1 h at 37 °C, then incubated overnight at 4 °C with primary antibodies. After TBST washes, membranes were probed with horseradish peroxidase (HRP)-conjugated secondary antibodies (1 h, 37 °C). Chemiluminescent detection was conducted using ECL substrate (TransGen Biotech, China) on a MicroChemi imaging system (DNR Bio-Imaging Systems, Israel). Band intensity quantification was performed using ImageJ 1.52 software (NIH, United States), normalized to GAPDH levels for statistical analysis. Primary antibodies included:INMT (Proteintech, 21578-1-AP), TGF-β (Proteintech, 21898-1-AP), SMAD4 (Proteintech, 10231-1-AP), β-catenin (Proteintech, 51067-2-AP), BAX (Proteintech, 60267-1-Ig), PARP1 (Proteintech, 13371-1-AP), BCL2 (Proteintech, 12789-1-AP), Caspase 3/P17/P19 (Proteintech, 19677-1-AP), GAPDH (Proteintech, 10494-1-AP). Antibody incubations were conducted following manufacturers’ protocols.

### Colony formation assay

The anti-proliferative effect of the drug was quantified by a colony formation assay. Cells were seeded at 500 cells per well in 6-well plates; after 24 h of drug exposure, culture medium was refreshed every 48 h until colonies became visible. Colonies were fixed with 70% ethanol, stained with crystal violet, rinsed twice with phosphate-buffered saline to remove excess dye, and enumerated.

### TUNEL assay

Cells plated in 24-well plates were exposed to the indicated agents for 24 h. After two PBS rinses, they were fixed in 4% paraformaldehyde for 20 min and washed twice with PBS. Membranes were permeabilized with 1% Triton X-100, followed by two PBS washes, and equilibrated for 15 min in equilibration buffer. The TdT reaction mix was then applied and the plates incubated at 37 °C for 1 h in the dark. Reactions were terminated with 2× SSC for 15 min, cells were washed twice with PBS, and nuclei were counterstained with DAPI.

### Intracellular reactive oxygen species (ROS) measurement

ROS content was evaluated with a commercial kit. After 24 h seeding at 1 × 10^5^ cells per well in 6-well plates, cultures were challenged with graded drug doses for a further 24 h. The cells were next exposed to 10 μM DCFH-DA for 30 min at 37 °C under light exclusion, rinsed with serum-free medium, and the resulting fluorescence was recorded on a Sony SA3800 spectral analyzer (λex 488 nm, λem 525 nm).

### Mitochondrial membrane potential (MMP) assessment

MMP was monitored with a JC-1 kit. Cells were plated at 1 × 10^5^ per well in 6-well plates, allowed to attach for 24 h, and then exposed to serial dilutions of the drug for an additional 24 h. After loading with JC-1 working reagent for 20 min at 37 °C and a single rinse with assay buffer, the resulting red/green fluorescence was quantified on a Sony SA3800 spectral analyzer.

### Migration and invasion analyses

Transwell assays were employed to evaluate both migration and invasion. For migration, 2 × 10^3^ cells were plated into the upper compartment of uncoated inserts; the lower well contained full medium. After attachment, both chambers were replenished with serum-free (upper) or complete (lower) medium supplemented with the indicated drug concentrations. After 24 h, non-migrated cells were removed with a cotton swab; those on the underside were fixed in 70% ethanol, stained with 0.1% crystal violet for 30 min, rinsed with PBS, dried, and counted under an Olympus IX73 microscope (100×) equipped with MShot software. Invasion assays followed the identical protocol except that the inserts were pre-coated with Matrigel.

### Wound-healing assay

Confluent monolayers generated by 24 h seeding in 6-well plates were linearly wounded with a sterile pipette tip, rinsed with PBS, and exposed to the drug. Images of the denuded area were acquired immediately and 24 h later, and gap closure was calculated with ImagePro Plus v6.1.

### RNA extraction and quantitative real-time PCR (qRT-PCR)

Total RNA was extracted with TRIzol® and reverse-transcribed using a commercial kit. Amplifications were run in triplicate with SYBR® Ex Taq™ Premix (Takara) on a real-time PCR platform; target values were normalized to GAPDH and calculated by the ΔΔCt method.

Primer Sequences (5′→3′):

GAPDH: F: GGA​GCG​AGA​TCC​CTC​CAA​AAT, R: GGC​TGT​TGT​CAT​ACT​TCT​CAT​GG.

INMT: F: TGG​AGA​AAG​AGG​AGG​TGG​AGC​AG, R:GGCAGCATTGGTGACAGAGTAGC.

### Transmission electron microscopy (TEM) sample preparation

Drug- or vehicle-treated cells were fixed with 2.5% glutaraldehyde and 1% OsO_4_, dehydrated through graded ethanols, and infiltrated with ethanol/Epon 812 mixtures (3:1, 1:1, 1:3). After resin embedding and ultramicrotomy, sections were stained with uranyl acetate (10 min) and lead citrate (2 min) at room temperature and examined in a JEOL JEM-1400FLASH electron microscope.

### Tumor xenograft model

BALB/c-nu/nu male mice (4–6 weeks; Vital River, Beijing) were maintained in a sterile SPF room at 24 °C ± 1 °C with unrestricted access to autoclaved food and water. All procedures were approved by the Medical Laboratory Animal Welfare and Ethics Committee of China Medical University and conducted in accordance with NIH guidelines. Animals received a single subcutaneous injection of 100 µL PBS containing 5 × 10^6^ 22RV1 cells into the right flank. Seven days later, when tumors were palpable, mice were randomly allocated to two groups (n = 6) and treated intraperitoneally every 3 days as follows: Group 1, vehicle (RPMI-1640); Group 2, DMPP-4M (10 mg kg^−1^). After 30 days of treatment (ten injections), mice were euthanized; tumors were excised and measured. Tumor volume was calculated with formula V = (length × width^2^)/2.

### Statistical analysis

All quantitative data are reported as mean ± SEM derived from a minimum of three independent biological replicates. Normality and homogeneity of variance were verified before formal testing. Comparisons between two independent groups of equal variance were performed with two-tailed unpaired Student’s t tests. Datasets containing more than two groups were analyzed by one-way analysis of variance (ANOVA) followed by Tukey’s *post hoc* multiple-comparison test. All computations were executed in GraphPad Prism version 9.0.0 (GraphPad Software, Inc., La Jolla, CA, United States). Probability values are displayed graphically as *p < 0.05, **p < 0.01, ***p < 0.001, and ****p < 0.0001; a threshold of p < 0.05 was considered statistically significant for every test performed.

## Results

### Efficient identification of INMT agonists

Initial computational analyses were conducted to identify potential ligand-binding sites on the INMT protein structure. A prominent putative binding pocket was identified distal to the catalytic domain. Molecular docking simulations suggest that occupancy of this allosteric site by small molecule ligands could induce conformational changes in the enzyme’s tertiary structure. This structural modulation may enhance INMT catalytic activity through a positive allosteric mechanism, potentially resulting in functional agonism of the enzyme.

### Protein structure acquisition and preprocessing

**FIGURE 1:**
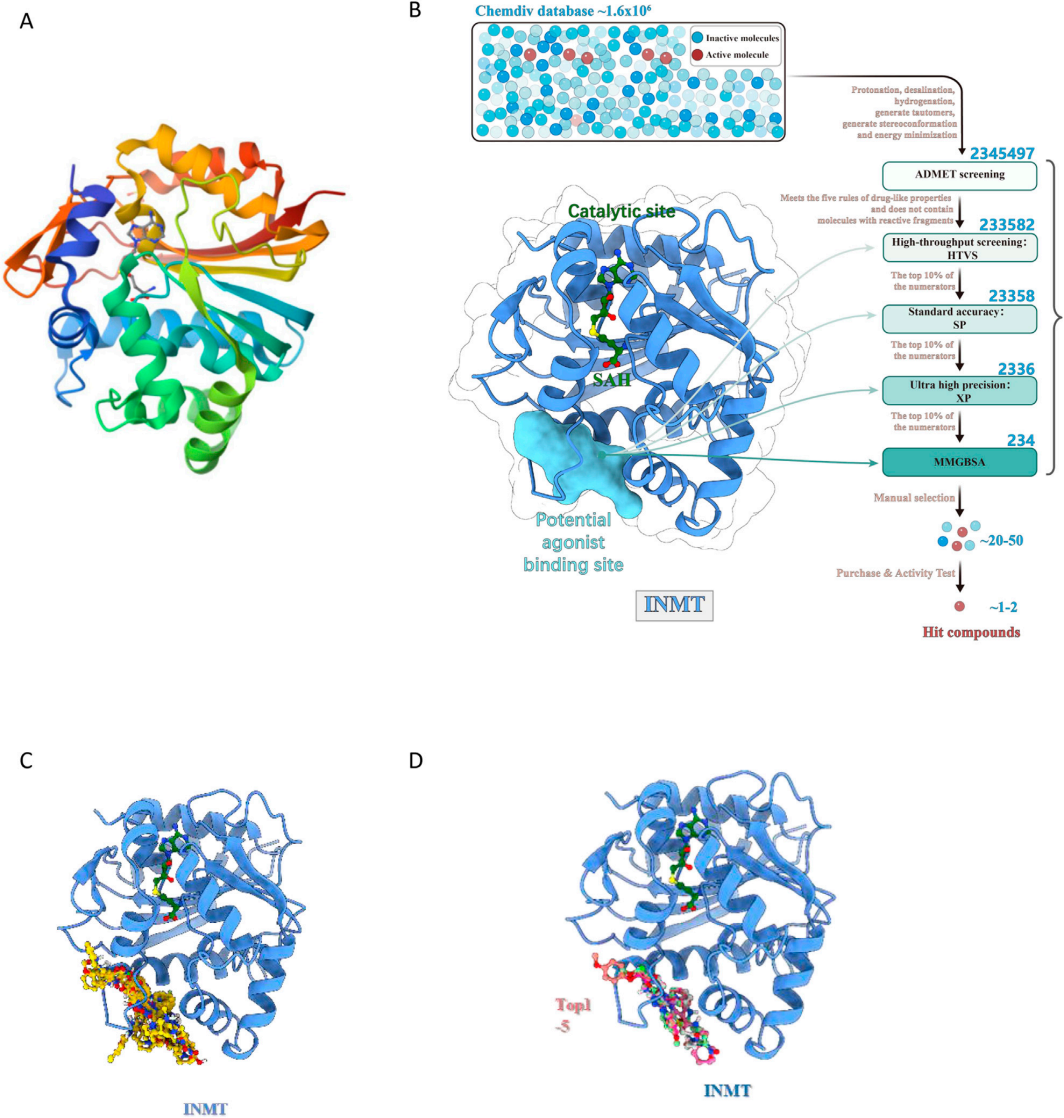
Protein structure of INMT and screening strategies for potential agonists. **(A)** Crystal structure of human indolethylamine N-methyltransferase with SAH. **(B)** Schematic diagram. **(C)** Structural superposition analysis of top 30 docking conformations. **(D)** Superposition of the top five docked molecules. Statistical significance: NS (not significant), *p < 0.05, **p < 0.01, ***p < 0.001, ****p < 0.0001.

The X-ray structure of INMT (PDB ID: 2A14) (Figure 1A) was obtained from the RCSB Protein Data Bank (https://www.rcsb.org/). The structure was then preprocessed using Schrödinger’s Protein Preparation module, which involved removing water molecules and extraneous ions, completing missing side chains and loops, and performing energy minimization. The SiteMap module was then used to predict the potential binding sites of INMT, and the Glide Grid module was used to generate a docking grid file for subsequent molecular docking studies.

### Small molecule database preprocessing

The screening database was obtained from ChemDiv (https://www.chemdiv.com/). The database was preprocessed using Schrödinger’s LigPrep module, which involved protonation, desalting, hydrogenation, generating tautomers and stereoisomers, and energy minimization.

### Virtual screening

The QuickProp module was used to perform an initial ADMET filter on the database molecules, retaining only those that met Lipinski’s five rules and did not contain reactive fragments. The Glide module was then used to perform the first round of virtual screening in high-throughput virtual screening (HTVS) mode. The top 10% of molecules from HTVS were advanced to standard precision (SP) docking, as illustrated in the tiered screening workflow (Figure 1B). Similarly, the top 10% of the molecules from SP screening were retained for final ultra-precision (XP) screening (Figure 1C). The top 10% of the molecules from XP screening were analyzed using MMGBSA and the binding free energy was calculated. Based on the MMGBSA scores, five molecules were selected for binding mode analysis.

### Molecular docking analyses characterized the binding interactions between small molecule compounds and the INMT protein

**FIGURE 2:**
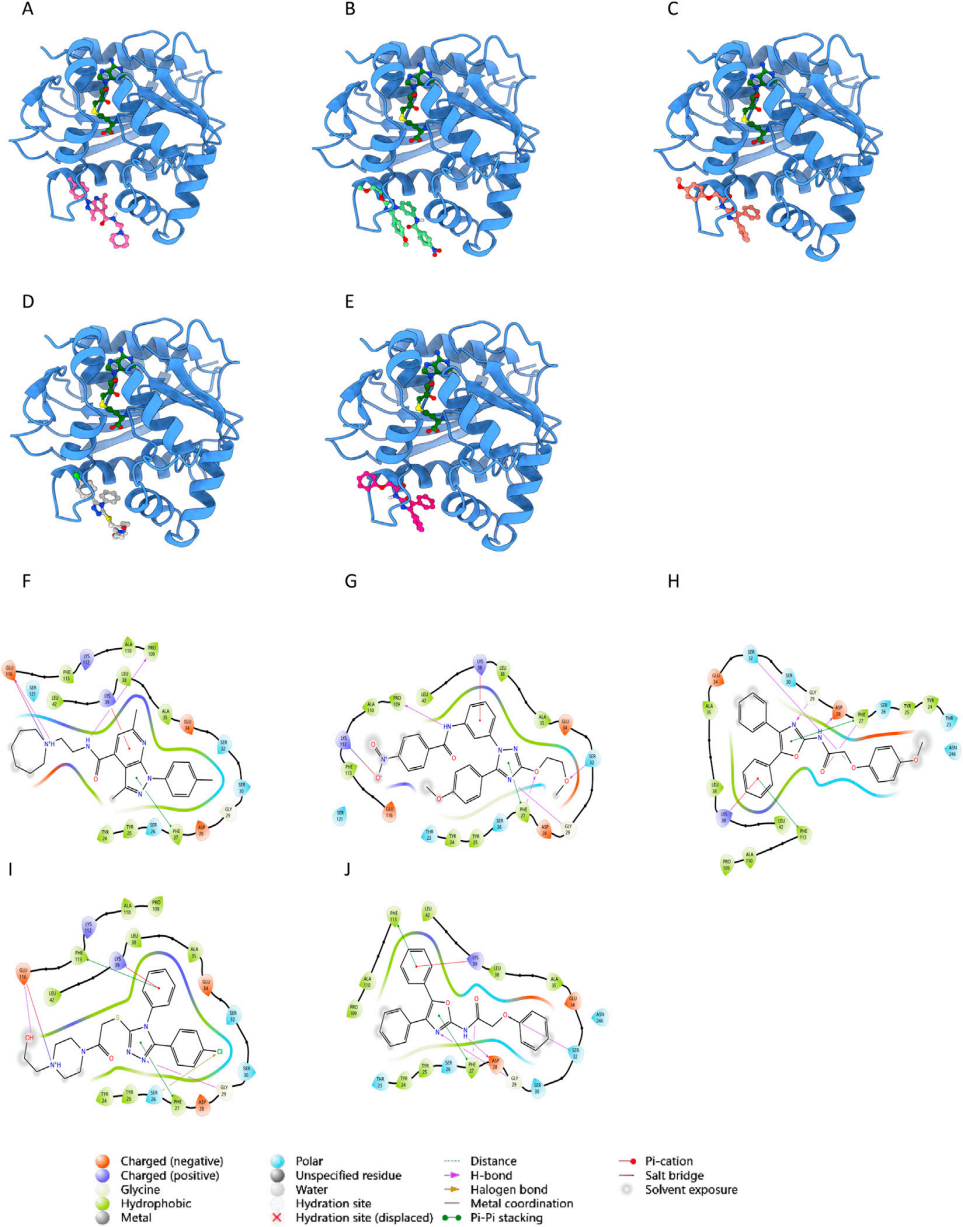
Docking results of small molecule compounds with INMT protein molecules. **(A–E)** Stereoscopic schematic of the top 1–5 small molecule bound to INMT. **(F–J)** 2D interaction diagram of the top 1–5 small molecule at the INMT binding site. Statistical significance: NS (not significant), *p < 0.05, **p < 0.01, ***p < 0.001, ****p < 0.0001.

The top 10% scoring molecules from XP screening underwent MMGBSA analysis to calculate binding free energy. Five compounds were selected for binding mode evaluation based on their MMGBSA scores. The chemical formulas and MMGBSA scores of these five compounds are presented (Table 1), and the specific binding conformations of these compounds to indolethylamine N-methyltransferase (INMT) are visualized (Figure 1D). Compound 1 occupied INMT’s putative allosteric activation site, forming hydrogen bonds, Pi-Pistacking, and salt bridge interactions with Phe27, Lys39, Pro109, and Glu116 near the catalytic region (Figures 2A,F). Compound Top2 occupied INMT’s putative allosteric activation site, forming hydrogen bonds, Pi-Pistacking, and salt bridge interactions with catalytic-adjacent residues Phe27, Gly29, Ser32, Lys39, Pro109, and Lys112 (Figures 2B,G). Compound Top3 targeted the same activation site, establishing hydrogen bonds, Pi-Pistacking, π-cation interactions, and salt bridges with Phe27, Asp28, Gly29, Ser32, Lys39, and Phe113 (Figures 2C,H). Compound Top4 engaged the activation pocket via hydrogen bonding, Pi-Pistacking, π-cation interactions, halogen bonding, and salt bridges involving Ser26, Phe27, Gly29, Lys39, Phe113, and Glu116 (Figures 2D,I). Compound Top5 demonstrated binding through hydrogen bonds, Pi-Pistacking, and π-cation interactions with Phe27, Asp28, Gly29, Ser32, Lys39, and Phe113 near the activation site (Figures 2E,J).

**TABLE 1:** MMGBSA scores for selected compounds.

MMGBSA dG bind	hit_id	Smile
−70.31	HIT211449390	Cc(c(c1nc(C)c2)c2C(NCCN2CCCCCC2)=O)nn1-c1ccc(C)cc1
−69.23	HIT102775992	COCCOc(nc1-c(cc2)ccc2OC)nn1-c1cccc(NC(c(cc2)ccc2[N+]([O-])=O)=O)c1
−68.49	HIT100265167	COc(cc1)ccc1OCC(Nc1nc(-c2ccccc2)c(-c2ccccc2)o1)=O
−68.1	HIT100409555	OCCN(CC1)CCN1C(CSc1nnc(-c(cc2)ccc2Cl)n1-c1ccccc1)=O
−67	HIT100070934	c1(nc(c(o1)c1ccccc1)c1ccccc1)NC(=O)COc1ccccc1

### DMPP-4M exhibits specific activation of INMT

**FIGURE 3:**
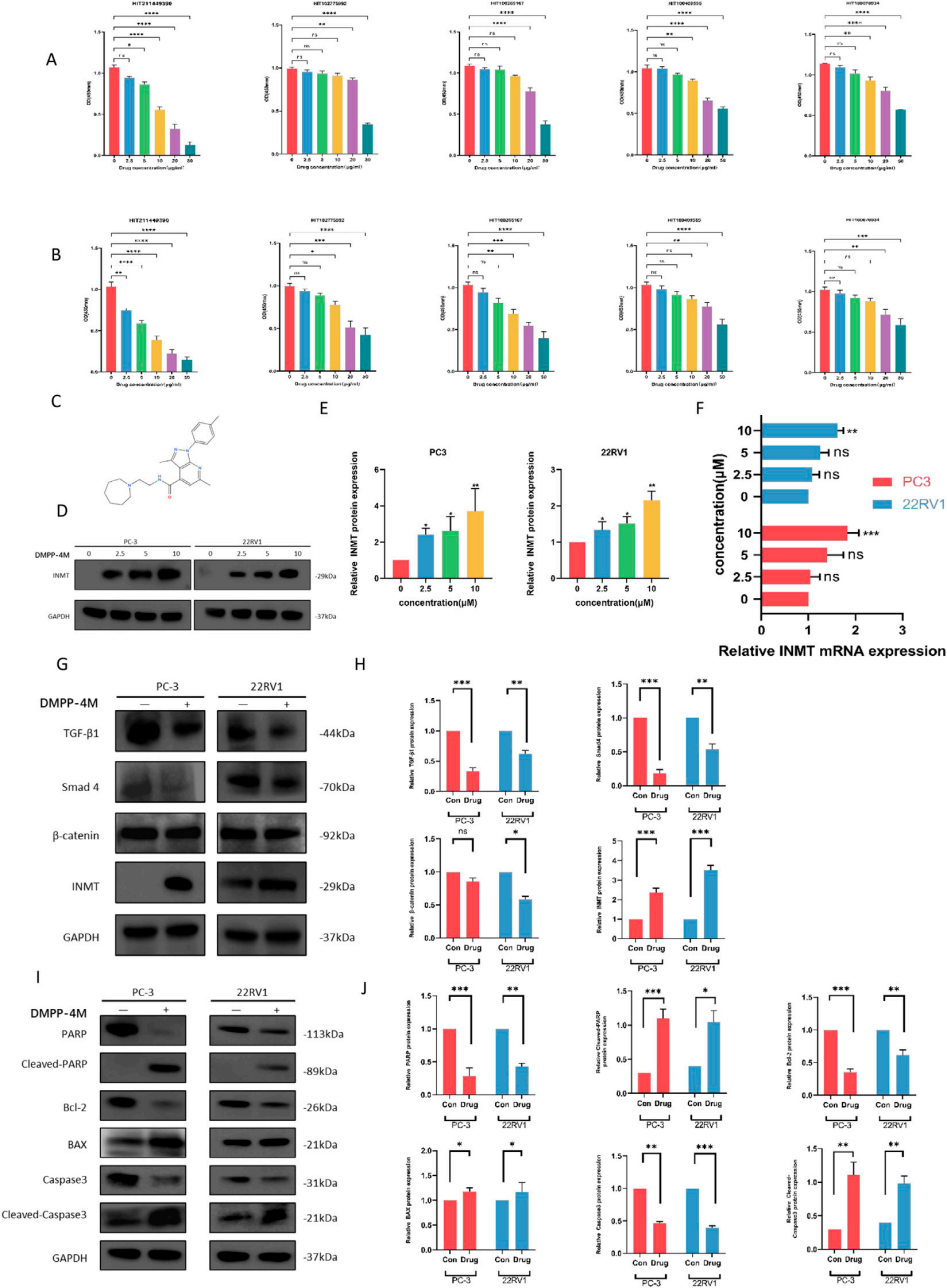
**(A)** The effect of small molecule compounds on PC-3 cell proliferation. **(B)** The effect of small molecule compounds on 22RV1 cell proliferation. **(C)** Chemical structure of DMPP-4M. **(D–F)** DMPP-4M upregulated the expression of INMT at both the protein and mRNA levels in a dose-dependent manner. **(G,H)** DMPP-4M modulates the Smad4, TGF-β1, and β-catenin signaling pathways. **(I,J)** DMPP-4M promotes the apoptosis of CRPC cells. Statistical significance: NS (not significant), *p<0.05, **p<0.01, ***p<0.001, ****p<0.0001.

The top five MMGBSA-ranked small molecules from prior screening were selected to validate their INMT binding affinity and activating efficacy. PC-3 and 22RV1 cells were exposed to graded concentrations of each compound for 48 h for quantitative assessment. All five compounds exhibited proliferation-inhibiting effects on both 22RV1 and PC-3 cells (Figures 3A,B). By calculating the IC_50_ of each compound against PCa cells (Table 2), Compound 1 was identified as the most potent cytotoxic agent among the five. Based on the structural formula retrieved from the compound library, this compound was designated as DMPP-4M (Figure 3C). Meanwhile, the results of Western blot (WB) and quantitative real-time polymerase chain reaction (RT-qPCR) assays demonstrated that DMPP-4M activates the transcription of INMT and increases the expression level of INMT protein (Figures 3D–F). Based on the experimental conclusions regarding the INMT signaling pathway from our previous studies, WB verified that after activating INMT, DMPP-4M regulates the expression levels of TGF-β1, Smad4 and β-catenin (Figures 3G,H). This is consistent with the conclusions of our prior research, ruling out the off-target effect of DMPP-4M.INMT can regulate the intrinsic apoptosis of tumor cells, and WB results verified that DMPP-4M promotes the intrinsic apoptosis of PCa cells (Figures 3I,J).

**TABLE 2:** IC_50_.

hit_id	HIT211449390	HIT102775992	HIT100265167	HIT100409555	HIT100070934
PC-3	9.9	33.6	36.1	65.3	50.6
RV1	8.5	34.1	29.5	61.2	85.1

### Molecular dynamics simulations characterized the interaction between DMPP-4M and the INMT protein

#### MD simulation analysis

**FIGURE 4:**
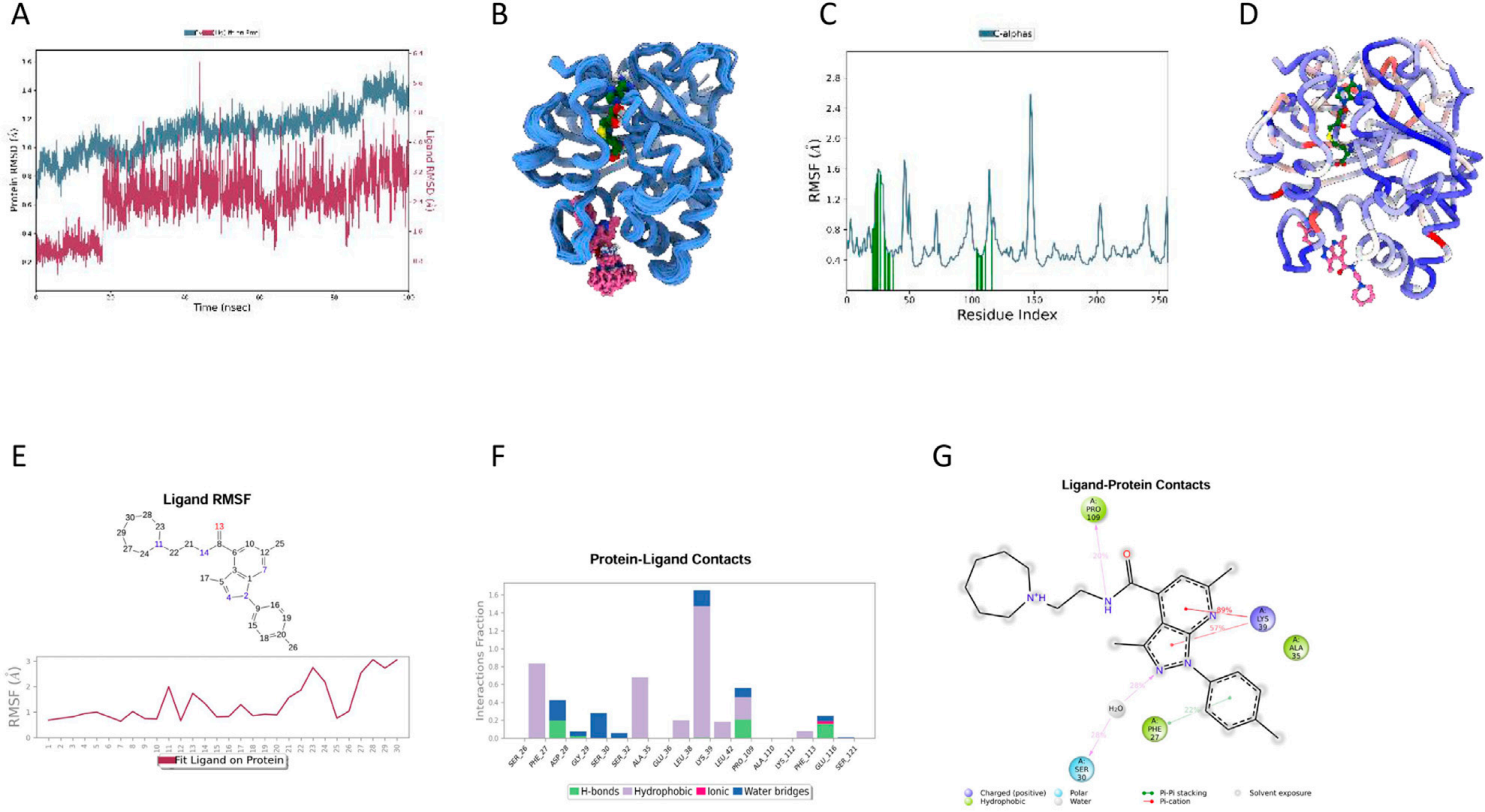
Molecular dynamics simulation results of DMPP-4M and INMT protein. **(A)** RMSD of protein (blue) and small molecule (red) during 100 ns molecular dynamics simulation. **(B)** Superposition of 100 snapshots sampled at 1 ns intervals during 100 ns MD Simulation. **(C)** RMSF of protein INMT during 20–100 ns MD simulation (Green highlights indicate residues interacting with the top 1 molecule). **(D)** B-factors derived from molecular dynamics (MD) trajectories. **(E)** Atom indices of the small molecule and its RMSF during 60–100 ns MD Simulation. **(F)** Contribution of binding site residues to small molecule binding. **(G)** 2D interaction diagram of the small molecule-protein binding mode. Statistical significance: NS (not significant), *p < 0.05, **p < 0.01, ***p < 0.001, ****p < 0.0001.

To further optimize the binding mode of INMT and the best-scoring small molecule, a conventional molecular dynamics (MD) simulation was performed on the protein-small molecule complex using the Desmond program. The OPLS2005 force field was used to parameterize the protein and small molecule, and the TIP3P model was used to solvate the water molecules. The protein-small molecule complex was placed in a cubic water box and dissolved, and then the system charge was neutralized by adding 0.150 M chloride and sodium ions. The system energy was minimized using steepest descent for 50,000 steps, followed by 50,000 steps of NVT and NPT equilibration, with the positions of the heavy atoms constrained. The system temperature and pressure were maintained at 300 K and 1 bar. After the two equilibration stages, an unconstrained 100 ns simulation was performed, with the energy and coordinates of the trajectory saved every 10 ps. Following 100 ns molecular dynamics simulations, trajectory analysis initiated with RMSD extraction for the INMT protein and DMPP-4M. RMSD plots demonstrated system stabilization within 20 ns, indicating structural equilibrium of the protein-ligand complex. Subsequent sampling focused on the 20–100 ns trajectory segment for detailed conformational analysis (Figure 4A). Conformational sampling at 1-ns intervals generated 100 trajectory snapshots during the 100 ns MD simulation. Structural superimposition of these conformers demonstrated high spatial congruence between protein and ligand configurations (Figure 4B). The ligand maintained stable binding within the active site throughout the simulation timeframe, as evidenced by consistent molecular alignment. Root mean square fluctuation (RMSF) analysis and B-factor calculations were performed on the INMT protein-ligand complex trajectory. The RMSF/B-factor profiles indicated limited structural flexibility across the protein (mean fluctuation <2.0 Å). Notably, a loop region adjacent to the ligand-binding site exhibited moderate conformational mobility. This localized flexibility suggests potential structural modulation upon ligand occupancy that may facilitate allosteric activation mechanisms (Figure 4C). Analysis of ligand RMSF profiles revealed minimal atomic fluctuations (<2.0 Å) in the Top1 molecule’s core region, indicating stable engagement within INMT’s binding pocket. The peripheral cavity region exhibited increased flexibility (Figure 4D), suggesting opportunities for structural optimization to enhance binding stability through targeted molecular modifications. Interaction analysis of the stabilized trajectory segment (20–100 ns) revealed key binding residues (Phe27, Ala35, Lys39, Pro109) critical for Top1 engagement (Figure 4E). These residues predominantly mediated binding through water-bridged interactions and hydrophobic contacts. Occupancy analysis of molecular interactions during the 20–100 ns trajectory revealed distinct binding patterns (Figure 4F). The internal cavity region exhibited frequent intermolecular contacts, while solvent-exposed regions showed negligible binding events. Top1 maintained stable binding through persistent hydrogen bonds between the ligand’s nitrogen atom and Ser30/Pro109 residues, complemented by a stable π-cation interaction between its aromatic moiety and Lys39. These coordinated interactions collectively stabilized the INMT-Top1 complex (Figure 4G).

#### DMPP-4M inhibits the proliferation, invasion, and metastasis of prostate cancer cells

**FIGURE 5:**
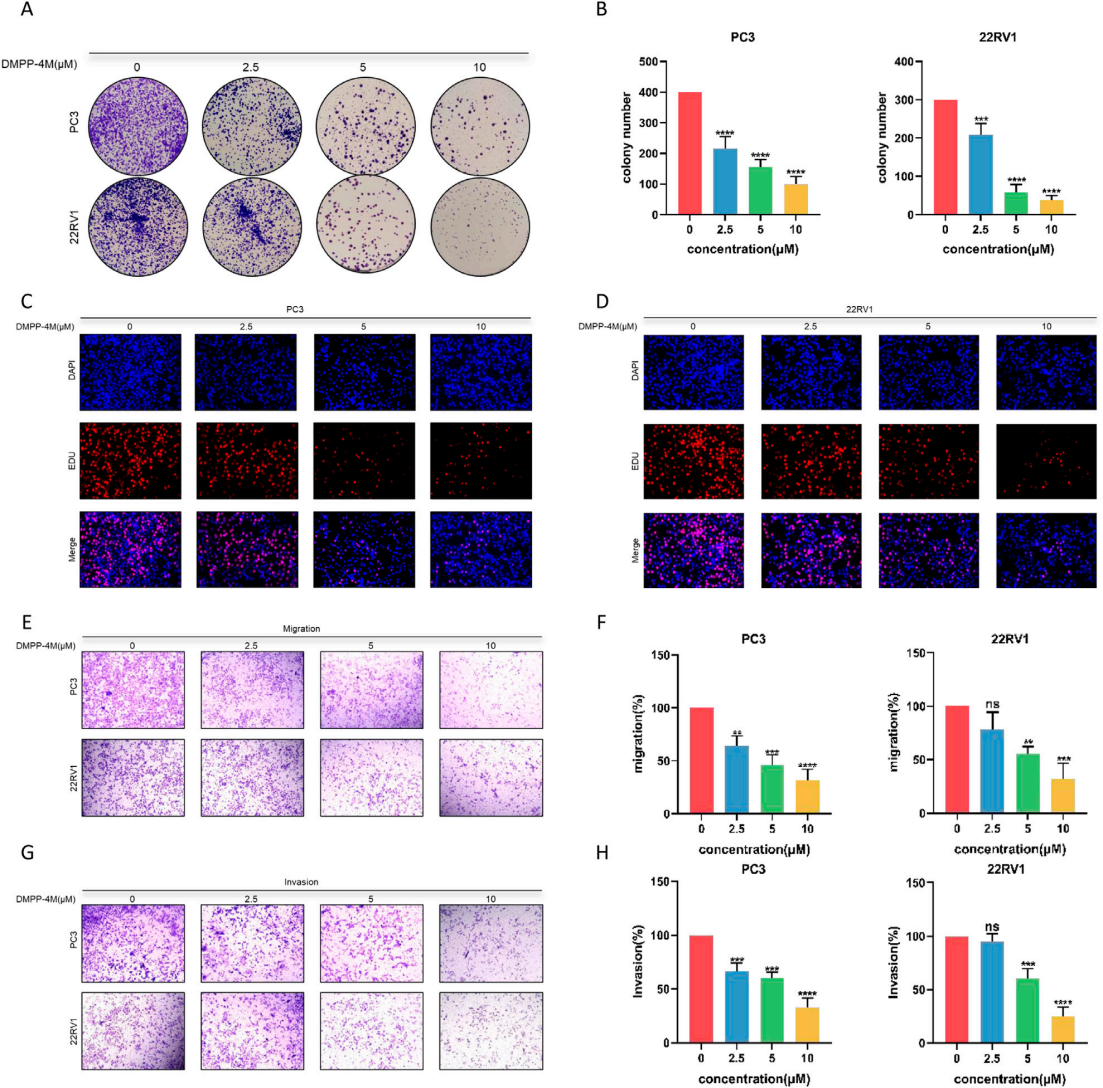
DMPP-4M inhibited the proliferation, migration, and invasion of CRPC cells in a concentration-dependent manner. **(A, B)** Colony formation assays demonstrated that DMPP-4M inhibited the proliferation of CRPC cells. **(C, D)** EdU incorporation assays demonstrated that DMPP-4M inhibited the proliferation of CRPC cells. **(E–H)** Transwell migration and invasion assays demonstrated that DMPP-4M inhibited the migration and invasion of CRPC cells. Statistical significance: NS (not significant), *p < 0.05, **p < 0.01, ***p < 0.001, ****p < 0.0001.

INMT by DMPP-4M exhibits a dose-dependent manner. The IC_50_ values of DMPP-4M in PC-3 and 22RV1 cells are 9.9 μM and 8.5 μM, respectively. Therefore, concentrations of 0, 2.5, 5, and 10 μM were selected for subsequent experiments in this study. Regarding the functional association between INMT and proliferation-related genes, in the cell colony formation assay, DMPP-4M reduced the average number of visible colonies in both PC-3 and 22RV1 cells (Figures 5A,B). In the EdU assay, DMPP-4M significantly decreased the proportion of proliferating cells (Figures 5C,D). These results indicate that DMPP-4M remarkably inhibits the proliferative capacity of PCa cells in a concentration-dependent manner. Tumor migration and invasion are crucial biological functions in the progression of CRPC. Therefore, this study investigated the effect of DMPP-4M on the migratory and invasive capabilities of PCa cells. Results from Transwell migration and invasion assays, showed that DMPP-4M significantly inhibited the migratory and invasive capabilities of the cells (Figures 5E–H). These results above indicate that DMPP-4M exhibits a concentration-dependent ability to inhibit the proliferation, migration, and invasion of PCa cells.

#### DMPP-4M promotes apoptosis in PCa cells via ROS

**FIGURE 6:**
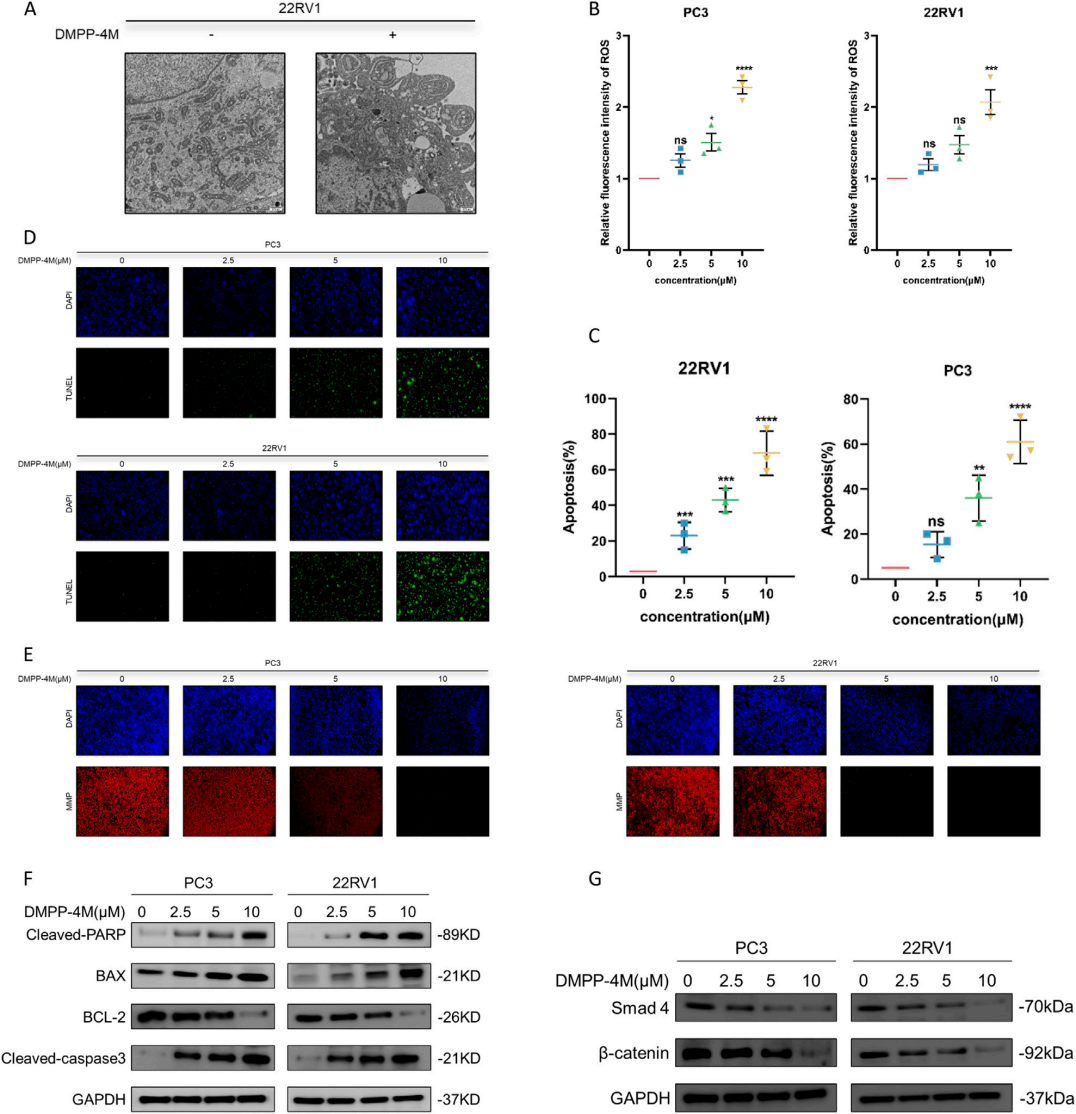
DMPP-4M induces intracellular ROS generation, which in turn causes mitochondrial damage and subsequent apoptotic cell death. **(A)** Mitochondrial swelling and damage were observed under TEM. **(B, C)** The ROS accumulation level and apoptotic cell ratio in CRPC cells treated with DMPP-4M were detected using a microplate reader with fluorescence detection. **(D)** The TUNEL assay was performed to detect the extent of cell apoptosis. **(E)** Fluorescent probes were used to detect the MMP level. **(F, G)** DMPP-4M promoted cell apoptosis in a concentration-dependent manner and modulated the related signaling pathways. Statistical significance: NS (not significant), *p < 0.05, **p < 0.01, ***p < 0.001, ****p < 0.0001.

Regarding the functional association between INMT and apoptosis-related genes, typical manifestations of mitochondrial damage were observed in 22RV1 cells treated with DMPP-4M under a transmission electron microscope (Figure 6A). The proportion of apoptotic cells and the accumulation level of ROS were significantly increased after drug treatment, as quantified by a fluorescence microplate reader (Figures 6B,C). This conclusion was also confirmed by TUNEL staining (Figure 6D). After the addition of DMPP-4M, the MMP of 22RV1 and PC-3 cells was significantly disrupted (Figure 6E). These results confirm that DMPP-4M promotes the intrinsic apoptosis of PCa cells. WB assays verified that DMPP-4M significantly altered the BAX/Bcl-2 ratio and increased the expression levels of Cleaved-caspase 3 and Cleaved-PARP (Figures 6F,G).

#### DMPP-4M inhibits the growth of PCa cell xenograft tumors *in vivo*

**FIGURE 7:**
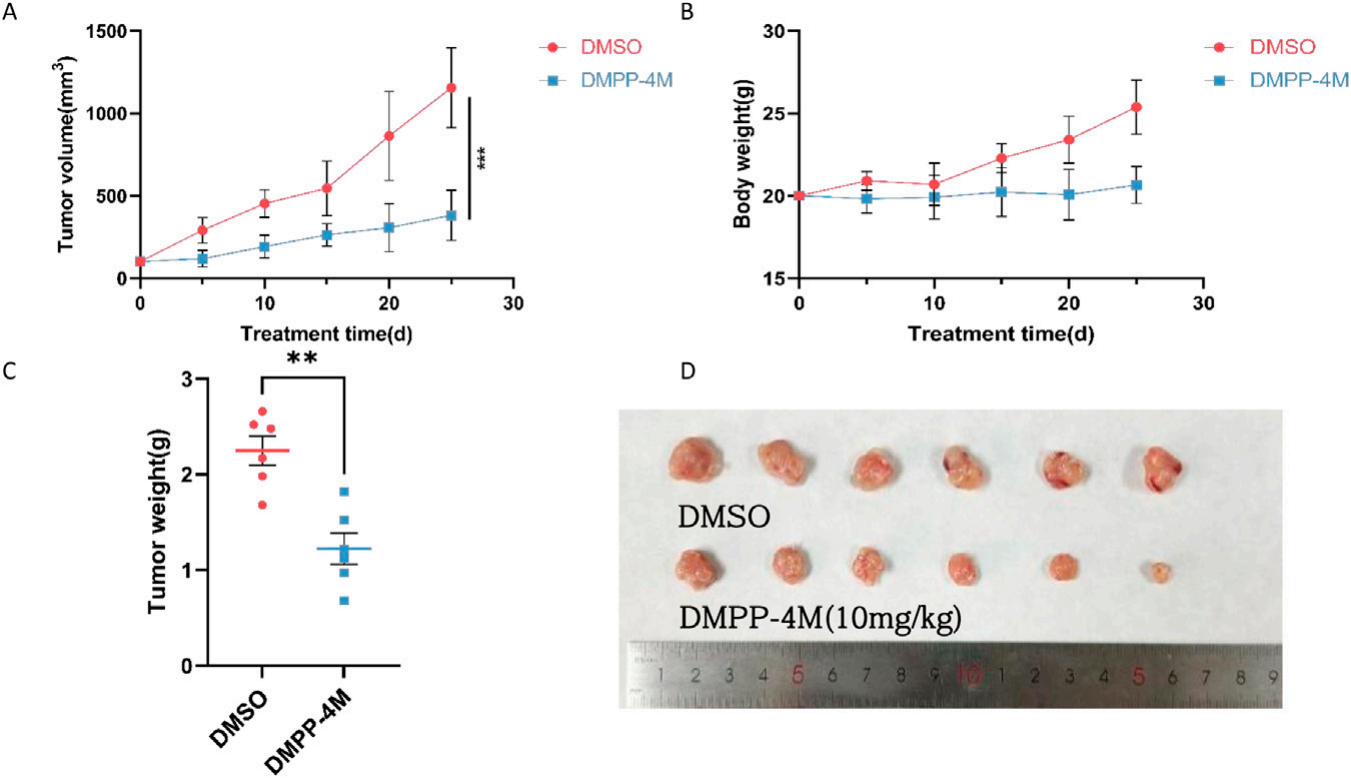
DMPP-4M suppressed tumor growth in xenograft mouse models. **(A)**Tumor volume measurements (n = 6). **(B)** Body weight changes during monotherapy and combination treatment (n = 6). **(C)** Tumor weight quantification (n = 6). **(D)** Representative tumor morphology variations (n = 6). Statistical significance: NS (not significant), *p < 0.05, **p < 0.01, ***p < 0.001, ****p < 0.0001.

To further investigate the inhibitory effect of DMPP-4M on tumor growth in a nude mouse xenograft model of PCa, we first evaluated the safety of the drug. Nude mice were intraperitoneally injected with DMPP-4M (10 mg/kg) every other day for 1 week, and no severe health issues or deaths were observed in the animals. Blood samples collected from the mice were subjected to blood biochemical analysis for liver and kidney function, and no abnormalities were detected. Meanwhile, the heart, liver, spleen, lung, and kidney tissues of the mice were subjected to hematoxylin-eosin (HE) staining, and no significant tissue necrosis was observed. Compared with the control group, the injection of DMPP-4M significantly inhibited the growth of tumor tissues in the animals (Figures 7A–D).

## Discussion

INMT, a pivotal enzyme catalyzing the methylation metabolism of tryptamine and related indole compounds (Chu et al., 2014; Jefford and Irminger-Finger, 2006), exhibits high expression in normal mammalian tissues including lung (Thompson and Weinshilboum, 1998), liver, and brain (Dean et al., 2019), while demonstrating marked downregulation in malignant tumors such as meningioma, lung cancer, and prostate cancer. Clinical analysis of prostate cancer specimens revealed significant inverse correlations between INMT expression levels and tumor metastasis (lymph node involvement), pathological grading (Gleason scores), and serum biomarker (PSA) concentrations (Jianfeng et al., 2022). This study employed the Schrödinger molecular docking platform to perform high-throughput virtual screening of the ChemDiv compound library based on the established INMT structure, successfully identifying multiple candidate molecules with potential for specific binding to INMT. Through Molecular Mechanics/Generalized Born Surface Area (MM/GBSA) binding free energy calculations, the top five candidate molecules exhibiting optimal binding energies were selected for interaction analysis at binding sites. During experimental validation, DMPP-4M was successfully identified via CCK-8 assays as a potent INMT agonist in both hormone-sensitive (22RV1) and hormone-resistant (PC-3) prostate cancer cell lines. Consequently, molecular dynamics simulations were conducted to investigate the dynamic binding pattern of DMPP-4M with INMT, providing theoretical guidance for subsequent structural modifications. Simulation results demonstrated that the compound formed stable interaction networks with the target protein, maintaining a root mean square deviation (RMSD) below 2.0 Å and achieving key hydrogen bond occupancy rates exceeding 85%.

The biological regulatory functions of INMT encompass critical biological processes including nuclear division regulation, chromosomal segregation processes, organelle fission mechanisms, and mitotic cell cycle transitions (Li et al., 2021). These processes participate in tumor progression mechanisms by modulating tumor cell proliferative activity, programmed death regulation, and genomic stability maintenance (Sun et al., 2007; Wagner and Nebreda, 2009). *In vitro* molecular validation experiments demonstrated that DMPP-4M elevated INMT protein expression in a dose-dependent manner via Western blot analysis. Administration of DMPP-4M in hormone-sensitive (22RV1) and castration-resistant (PC-3) prostate cancer cell lines induced marked upregulation of BAX protein expression, significant downregulation of Bcl-2 levels, and dose-dependent elevation of cleaved Caspase-3 and cleaved-PARP fragments concomitant with increased total protein content. These findings collectively demonstrate that DMPP-4M activates INMT expression, suppresses CRPC cell proliferation, and induces apoptosis via an AR-independent mechanism in CRPC models.

INMT modulates apoptotic processes through regulation of multiple tumor-associated signaling pathways. Research demonstrates that TGF-β, functioning as a pleiotropic cytokine, exerts pathophysiological effects encompassing cellular migration control, matrix adhesion modulation, proliferation suppression, and differentiation induction (Ikushima and Miyazono, 2010; Gelmann, 2002). The Smad-dependent signaling pathway exhibits dual-phase regulatory roles during tumor progression: during early stages, it exerts tumor-suppressive effects by inhibiting cell cycle progression and inducing apoptosis, whereas in advanced malignancies, oncogenic mutations inactivate its tumor-suppressive capacity, subsequently driving epithelial-mesenchymal transition (EMT) and enhancing tumor cell invasiveness and metastatic potential (Kypta and Waxman, 2012; Dehm and Tindall, 2006). Western blot analysis revealed that INMT negatively regulates the TGF-β/Smad signaling pathway, with particularly pronounced inhibitory effects on Smad4 expression in castration-resistant prostate cancer cells (PC-3). The canonical Wnt/β-catenin signaling pathway, serving as a central regulatory hub for prostate cancer cell proliferation, differentiation, and invasive phenotypes, demonstrates signaling efficacy closely associated with nuclear translocation levels of β-catenin (Kypta and Waxman, 2012). Mechanistic investigations suggest that INMT potentially mediates crosstalk between tumor metabolic reprogramming and signaling networks through modulation of core effector molecules including β-catenin. The mechanisms by which INMT activation suppresses TGF-β/Smad and Wnt/β-catenin signaling likely involve multiple pathways. First, as a methyltransferase, INMT may directly methylate key signaling proteins such as Smad4 and β-catenin, regulating their stability and nuclear translocation. Second, INMT may exert indirect effects through metabolic reprogramming, as its involvement in tryptophan metabolism and redox homeostasis can influence cellular signaling through AMPK or mTOR pathways. Third, INMT may regulate these pathways via MAPK crosstalk, given our previous finding that INMT modulates MAPK signaling. Future proteomic and metabolomic studies will be needed to identify the direct substrates of INMT and fully elucidate the mechanistic details.

Comparative analysis with existing research reveals that this study represents the first attempt to integrate INMT agonist development with CRPC therapeutics. Previous investigations predominantly focused on INMT’s roles in neurotransmitter metabolism and selenocompound detoxification, while its mechanistic involvement in tumor biology remained poorly characterized (Kuehnelt et al., 2015; Shibata et al., 2015). Compared with current CRPC therapies, DMPP-4M offers the advantage of an AR-independent mechanism, which may overcome resistance associated with AR-targeted agents such as enzalutamide and abiraterone. Additionally, DMPP-4M demonstrated good tolerability *in vivo*, in contrast to conventional chemotherapies like docetaxel that often cause significant systemic toxicity. This investigation employed molecular docking and dynamic simulations to elucidate the binding mode of DMPP-4M with INMT, demonstrating its stable interaction with an allosteric region adjacent to the catalytic site through hydrogen bonding and Pi-cation interactions. These structural engagements may facilitate enzymatic activation via conformational modulation, thereby establishing a critical framework for subsequent agonist optimization. The identification of this allosteric binding mechanism provides valuable insights for rational structural refinement of INMT-targeted therapeutic agents.

This investigation presents certain limitations that should be acknowledged. First, the *in vivo* efficacy study was conducted using only a single dose of DMPP-4M (10 mg/kg) without comprehensive dose-response characterization or a positive control group. While our *in vitro* experiments consistently demonstrated dose-dependent effects across multiple functional assays (CCK-8, colony formation, apoptosis, and Western blot), a full *in vivo* dose-response study would provide more complete pharmacological information. Additionally, head-to-head comparison with clinically approved CRPC agents (e.g., enzalutamide, docetaxel) would better benchmark the therapeutic potential of DMPP-4M. These studies are important next steps that we are actively planning for our follow-up work. Second, their potential to reverse the drug resistance of CRPC and to be combined with multiple clinically approved drugs remains to be explored. The precise molecular mechanisms underlying INMT activation—such as alterations in methylated substrates and their downstream signaling consequences—remain incompletely elucidated. Additionally, potential off-target effects of DMPP-4M on other signaling pathways (e.g., MAPK) require further investigation. INMT exerts distinct regulatory mechanisms across different CRPC cell lines. This variability is likely associated with genomic alterations, activation of AR splice variants, and engagement of bypass signaling pathways in CRPC, and this research direction warrants further exploration and elaboration. Future studies should validate the *in vivo* efficacy of the drug using patient-derived xenograft (PDX) animal models, and characterize the substrate methylation and other protein modifications induced by INMT activation through metabolomic and proteomic analyses. Concurrently, chemical modifications targeting peripheral flexible regions of the binding site may enhance compound selectivity and potency.

## Data Availability

The original contributions presented in the study are included in the article/supplementary material, further inquiries can be directed to the corresponding authors.
